# The multiple personalities of Watson and Crick strands

**DOI:** 10.1186/1745-6150-6-7

**Published:** 2011-02-08

**Authors:** Reed A Cartwright, Dan Graur

**Affiliations:** 1 Department of Biology and Biochemistry, University of Houston, Houston, Texas 77204-5001, USA

## Abstract

**Background:**

In genetics it is customary to refer to double-stranded DNA as containing a "Watson strand" and a "Crick strand." However, there seems to be no consensus in the literature on the exact meaning of these two terms, and the many usages contradict one another as well as the original definition. Here, we review the history of the terminology and suggest retaining a single sense that is currently the most useful and consistent.

**Proposal:**

The *Saccharomyces *Genome Database defines the Watson strand as the strand which has its 5'-end at the short-arm telomere and the Crick strand as its complement. The Watson strand is always used as the reference strand in their database. Using this as the basis of our standard, we recommend that Watson and Crick strand terminology only be used in the context of genomics. When possible, the centromere or other genomic feature should be used as a reference point, dividing the chromosome into two arms of unequal lengths. Under our proposal, the Watson strand is standardized as the strand whose 5'-end is on the short arm of the chromosome, and the Crick strand as the one whose 5'-end is on the long arm. Furthermore, the Watson strand should be retained as the reference (plus) strand in a genomic database. This usage not only makes the determination of Watson and Crick unambiguous, but also allows unambiguous selection of reference stands for genomics.

**Reviewers:**

This article was reviewed by John M. Logsdon, Igor B. Rogozin (nominated by Andrey Rzhetsky), and William Martin.

## Background

In 1953, James Watson and Francis Crick published the structure of DNA [1], for which they were awarded a Nobel Prize in 1962. They determined that DNA consists of two antiparallel, complementary strands twisted around each other to form a right-handed double helix held in place by interactions between complementary base pairs: adenine (A) with thymine (T) and guanine (G) with cytosine (C). From this structure, it was straightforwardly evident how the genetic information was copied and maintained [2].

As a couple, Watson and Crick were immediately hyphenated and eponymized, resulting in terms such as "Watson-Crick model" [3], "Watson-Crick structure" [4], "Watson-Crick helix" [5], "Watson-Crick duplex" [6], "Watson-Crick hydrogen bond" [7], "Watson-Crick bridge" [8], "Watson-Crick complementarity" [5], as well as "Watson-Crick base pair" [9] and its antonym "non-Watson-Crick base pair" [10]. These terms are unequivocal and easily understood. Interestingly, the eponym "Watson-Crick" has even been coopted by outsiders, such as in the field of formal languages, e.g. "Watson-Crick D0L system" [11]; although in a significant minority of non-biological allusions, the order of the names tends to be reversed as "Crick-Watson" instead of "Watson-Crick" [12,13].

In contrast to the clarity of the "Watson-Crick" modifier, the individual fates of the "Watson" and "Crick" eponyms turned out to be a terminological nightmare. In the literature, it has become popular to refer to the two strands of DNA as the "Watson" and "Crick" strands (sometimes abbreviated as W and C). However, it is not clear which strand is which, and the literature abounds in contradictory uses (Table 1).

**Table 1:** Watson- and Crick-strand definitions

Definition	Watson	Crick
Original	cytosine-poor	cytosine-rich
Compositional	pyrimidine-rich	purine-rich
Transcriptional	antisense	sense
Replicational	lagging	leading
Arbitrary	this	that
Database	top/plus	bottom/minus
5' to 3'	left to right (top or left-hand)	right to left (bottom or right-hand)

The earliest reference that we could find to the "Watson strand" and the "Crick strand" is somewhat tongue-in-cheek and comes from a pair of papers in 1967 by Wacław Szybalski and colleagues [14,15]. They bound the two DNA strands of phage λ to the synthetic polynucleotide, poly(IG), which has an affinity to cytosine-rich regions. They then separated the two strands by density, which turned out to be determined by the amount of bound poly(IG). In a cesium-chloride density gradient, the strand with more bound poly(IG) was denser and heavier than its complement. Because the "dense" strand was cytosine-rich, Szybalski and colleagues called it the "C strand." Logically, thus, the complementary strand, which was guanine rich, should have been the "G strand." Instead, it was christened the "W strand."

Intriguingly, the names of Watson and Crick are not mentioned explicitly. In time, the "W strand" and the "C strand" acquired unabbreviated names, "Watson strand" and "Crick strand," respectively [16]. Interestingly, in the absence of poly(IG), the "dense" C strand had a lower molecular weight than the "light" W strand. Thus, the lexicographic journey of the Watson and Crick strands started with the former denoting the light strand and the latter denoting the heavy strand. In time, the presence of the poly(IG) molecule would be forgotten and the definition of the two strands would reverse. Crick became the heavy, purine-rich strand, and Watson became the light, pyrimidine-rich strand [17].

In the literature, the original definition and its inverse are infrequently used today. When searching for either "Watson strand" or "Crick strand" through Google, the *Molecular Biology Glossary *at Chang Bioscience is currently the top hit [18]. This glossary defines the Watson strand as the antisense strand for transcription and the Crick strand as the sense strand. This usage is not only restricted to the online glossary but is also found in the scientific literature [19,20]. Other authors have used Watson and Crick strands in the context of DNA replication, (e.g. [21]) with the Watson strand denoting the lagging strand and the Crick strand denoting the leading strand. Sometimes the Watson and Crick strands are used as arbitrary labels, equivalent to "this strand" and "that strand" [22-24]. Without exception, in all cases in which the two strands are drawn horizontally in a figure, the 5' to 3' sequence on top is called the Watson, and the complementary 3' to 5' sequence at the bottom is designated the Crick [14,15,19,22,24-26]. In those rare cases in which the two strands are drawn vertically, the Watson strand is invariably the left-hand strand and Crick is the right-hand one.

**Figure 1:**
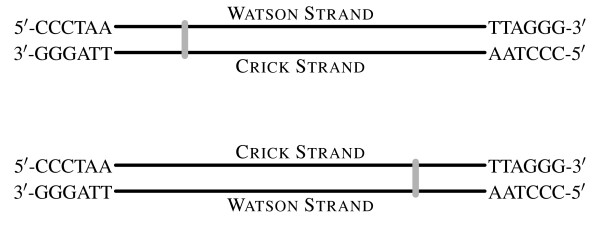
**Standardized definitions for Watson and Crick strands**. *Sensu stricto*, a genomic reference is used, like a submetacentric centromere (gray bar), to define two unequal arms of a chromosome. The Watson strand is the strand of a chromosome that has its 5'-end at the short-arm telomere and its 3'-end at the long-arm telomere. The Crick strand is the strand of that has its 5'-end at the long-arm telomere and its 3'-end at the short-arm telomere. If a chromosome is oriented differently, the designations still apply, providing much needed terminological consistency. The Watson strand should be stored as the reference (+) strand in a genomic database. Usage of the terms "Watson strand" and "Crick strands" are discouraged outside of a genomic context. If no genomic reference is possible, then it is acceptable to use these terms *sensu lato*, where the Watson strand is simply a database's reference strand, and the Crick strand its complement.

Arguably the most popular usage today originated with the *Saccharomyces *Genome Database (SGD), which defines the Watson strand as the strand which has its 5'-end at the left telomere and the Crick strand as its complement [25]. The left telomere is defined based on the pre-genomics linkage maps. In yeast genetics, the short arm of a chromosome was consistently chosen to be the "left" arm in these maps (EL Hong, personal communication; Figure 1). (Note: the left arms were placed above the centromere if the linkage maps were drawn vertically.) SGD uses the Watson strand as the forward, top, reference strand (+) in their database. The assignment of Watson to the top strand is not arbitrary but rather a reflection of the horizontal drawing convention. This yeast-genome terminology has been partially picked up by other genomicists, e.g. [27].

The *Saccharomyces *Genome Database utilizes the Watson-strand and Crick-strand designations to assign every gene a systematic name based on its position in the S. *cerevisiae *genome [25]. For example, the alcohol dehydrogenase I (ADH1) gene was assigned the systematic name YOL086C, and the enolase I (ENO1) gene has the systematic name YGR254W. These names begin with a letter denoting the organism, in this case "Y" for yeast, followed by the letters "A" to "P" for chromosomes I to XVI. Next "L" is used to denote the short (left) arm, and "R" the long (right) arm. A three-digit number denotes the ordinal position as counted from the centromere. Finally, "W" and "C" indicate whether the gene is located on the Watson or Crick strands, respectively. Thus, the systematic name for ADH1, YOL086C, means that the gene is found on chromosome XV, that it is the 86th gene from the centromere on the short arm, and that it is encoded on the Crick strand. Similarly, YGR245W means that enolase I is on chromosome VII, that it is the 245th gene from the centromere on the long arm, and that it is encoded on the Watson strand.

## Discussion

We believe that the existence of competing and contradictory usages of Watson and Crick strands leads to confusion, especially as scientific publications become more and more integrated with automated databases. For instance, a DNA sequence may concurrently be a Crick (sense) strand, a Watson (light) strand, a Crick (leading) strand, and may be located on the Watson (genomic) strand. This confusion of terminology will cause problems for automated literature mining. Biological research has become so vast that the ability of individuals to keep up with the literature relevant to their research has reached a breaking point [28,29]. In order to cope with the information explosion, scientists are starting to utilize software that automates the discovery of relevant peer-reviewed literature. The development of such software is an active area of research in bioinformatics and computational linguistics [28,29]. Such techniques are predicated upon the existence of unambiguous scientific terminology.

Can we standardize the terms "Watson strand" and "Crick strand"? In biology, the principle of precedence or "original intent" is sometimes used to decide among competing terminologies. This is certainly the case in taxonomy, in which, with few exceptions, the valid name for a species is the first name that was published, and the rest are invalid "junior synonyms." These are the rules that required school children to replace their much-loved *Brontosaurus *with the despicable *Apatosaurus*, the valid senior synonym [30]. In the case of the strand terminology, this principle would dictate the use of the least common and least useful sense in the literature. We propose instead to use the terms "Watson strand" and "Crick strand" in the sense developed by yeast genomicists and used by other eukaryotic genome projects. Not only is this usage consistent and useful, but gene names and genomic locations often rely on them.

Given the amount of effort already spent on standardizing such databases, and their influence on other disciplines, we feel that the genomic definition of Watson and Crick strands has the most mass behind it. Specifically, we find that the unambiguous usage of the *Saccharomyces *Genome Database to be the most useful. Under the first part of our proposal (Figure 1), the centromere is a reference point that divides a chromosome into two arms of unequal length. The chromosome is oriented so that shorter arm is on the left and the longer arm on the right. Furthermore, the top strand has its 5'-end at the left (short-arm) telomere and its 3'-end at the right (long-arm) telomere. This strand is the Watson strand. Similarly, the bottom strand has its 5'-end at the right telomere and its 3' at the left telomere and is the Crick strand. We further propose that "top", "forward", and "plus" be used as synonyms for the Watson strand and "bottom", "reverse", and "minus" for the Crick strand.

We note, however, that this suggestion does not provide a universal solution to all double-stranded DNAs; it deprives prokaryotes, centromere-less chromosomes, chromosomes with multiple centromeres, as well as double-stranded DNA viruses of their Watson and Crick strands, and does not even touch upon the problem of triple-stranded DNA, with its Watson, Crick and Hoogsteen strands [31]. In many of these situations, a genomic feature other than a centromere can be used to orient chromosomes unambiguously. For circular chromosomes, the origin of replication may be used in place of the centromere, while the location of termination can define a cutting point to create short and long arms. If it is ultimately impossible to distinguish Watson and Crick strands using biological properties, then we propose that Watson should refer to the stand arbitrarily used as a reference in a database (i.e. the "plus" stand) and the Crick strand should refer to its complement (Figure 1). With this two level approach, our proposal offers a nearly universal solution for unambiguously using Watson and Crick stand terminology, which should improve clarity and annotation.

## Competing interests

The authors declare that they have no competing interests.

## Authors' contributions

Both RAC and DG conceived and wrote the article.

## Reviewers Comments

### Reviewer 1

John M. Logsdon, Jr., Department of Biology, University of Iowa, Iowa City, IA 52242 USA

This is an interesting paper that makes a single important suggestion that I readily endorse. The historical backdrop that the authors develop as grist for the recommendation is in itself a worthwhile and enjoyable read.

### Reviewer 2

Igor B. Rogozin, NCBI/NLM/NIH, nominated by Andrey Rzhetsky

I am not an expert in scientific terminology. Thus I will discuss my personal experience and cannot guarantee that my opinion is correct. I think that personal names should be attached to theorems/equations/laws/rules/models (in other words, theoretical constructs) rather than to biological objects. For example, the term "Rogozin hotspots" is used sometimes to define mutable motifs associated with hotspots of somatic mutations in immunoglobulin genes (for example, Faili A, Aoufouchi S, Guéranger Q, Zober C, Léon A, Bertocci B, Weill JC, Reynaud CA. AID-dependent somatic hypermutation occurs as a DNA single-strand event in the BL2 cell line. Nat. Immunol. 2002, V.3, N 9, 815-821). This is Ok because this terms stands for a generalized rule (a consensus sequence RGYW/WRCY in this case). However, this term cannot be used for a fragment of DNA which contains this mutable motif because this is a biological sequence (object).

If we apply the same logic to double-stranded DNA, than the double helix of DNA is a model and we are in a position to denote it the Watson-Crick DNA model. However, I do not think that it is a good idea to assign names of people to DNA strands as these strands are biological objects. Sometimes DNA is single-stranded, and in this case the logic proposed by the authors cannot be applied. In the case of the yeast genome, I would prefer to use terms "direct" or "complementary" strand (and, accordingly, "D" and "C" instead of "W" and "C" in the name of genes) because it will be easier to immediately interpret these names. Of course, one needs to keep in mind that some traditional names of biological entities are inseparably linked to the names of their discoverers (e.g., the Golgi complex or Cajal bodies), and no one suggests renaming these, but I believe that propagation of this tradition requires a lot of caution and could be even counter-productive.

**Authors' Comments**: *We regard eponyms--terms based on or derived from a person's name--much more positively than Dr. Rogozin, whether they are applied to concepts or material entities. In fact, as we are from the University of Houston (an eponym) in the United States of America (another eponym), we would like to encourage the use of eponyms as a celebration of scientists and their work. The dictionary abounds in names of people attached to biological "objects" from cells (Leydig cells, islets of Langerhans), organs (Müllerian ducts, Darwin's tubercle), diseases (Tay-Sacchs), and DNA sequences (Hogness-Goldberg box), to viruses (Epstein-Barr), plants *(Banksia, Pointsettia), *and animals *(Drosophila willistoni). *Nomenclature in science should be exact, unambiguous, and if possible, pronounceable; no additional caution is necessary for eponymous nomenclature*.

### Reviewer 3

William Martin, University of Duesseldorf

This is an interesting, worthwhile, and scholarly paper. I think it should be published, but I have a request. Can the authors suggest a convention for circular chromosomes and plasmids based on origins of replication (oris)?

**Authors' Comments**: *In revision, we propose that the origin of replication and the location of termination can be used instead of the centromere and the telomeres, respectively. However, this might not be sufficient if the location of termination is evenly spaced from the origin of replication. In addition, there appears to be much variation in the nature of origination and termination of replication on circular chromosomes, and our proposal is probably not nuanced enough to handle every case*.

But I bet we can find yeast linkage maps where the chromosomes are drawn as vertical lines (short hen top?). It might be interesting to find out when and why that convention was chosen, or to point out that the authors could not find out whence it came (maybe a learned reader will enlighten us). How did Morgan draw chromosomes ...? Does someone from the yeast genomics community know how the W-C convention started (B Dujon)?

**Authors' Comments**: *Our knowledge about yeast linkage maps comes from a personal communication from EL Hong, Scientific Curator at SGD. She pointed us to the "Mortimer" maps (*http://www.yeastgenome.org/community/mortimer_maps/edition12.shtml*). Edition 12 drew the chromosomes vertically, with the "left" arms of the chromosomes above the centromere. A quick survey of previous editions found the earliest maps to be horizontal with the shorter arms drawn as the left chromosomes. However, no direct explanation of the convention was found*.
